# Glucose-dependent anaplerosis in cancer cells is required for cellular redox balance in the absence of glutamine

**DOI:** 10.1038/srep32606

**Published:** 2016-09-08

**Authors:** Naniye Mallı Cetinbas, Jessica Sudderth, Robert C. Harris, Aysun Cebeci, Gian L. Negri, Ömer H. Yılmaz, Ralph J. DeBerardinis, Poul H. Sorensen

**Affiliations:** 1 Department of Pathology and Laboratory Medicine, University of British Columbia, Vancouver, BC V5Z 1L3 Canada; 2 Department of Molecular Oncology, British Columbia Cancer Research Centre, Vancouver, BC V5Z 1L3, Canada; 3 Children’s Medical Center Research Institute, University of Texas-Southern Medical Center, Dallas, TX 75390-9063, USA; 4 The David H. Koch Institute for Integrative Cancer Research at MIT, Cambridge, MA 02139, USA; 5 Abdullah Gul University, Faculty of Engineering, 38170, Kayseri, Turkey; 6 Department of Biology, MIT, Cambridge, MA 02139, USA; 7 Department of Pathology, Massachusetts General Hospital and Harvard Medical School, Boston, MA 02114, USA

## Abstract

Cancer cells have altered metabolism compared to normal cells, including dependence on glutamine (GLN) for survival, known as GLN addiction. However, some cancer cell lines do not require GLN for survival and the basis for this discrepancy is not well understood. GLN is a precursor for antioxidants such as glutathione (GSH) and NADPH, and GLN deprivation is therefore predicted to deplete antioxidants and increase reactive oxygen species (ROS). Using diverse human cancer cell lines we show that this occurs only in cells that rely on GLN for survival. Thus, the preference for GLN as a dominant antioxidant source defines GLN addiction. We show that despite increased glucose uptake, GLN addicted cells do not metabolize glucose via the TCA cycle when GLN is depleted, as revealed by ^13^C-glucose labeling. In contrast, GLN independent cells can compensate by diverting glucose-derived pyruvate into the TCA cycle. GLN addicted cells exhibit reduced PDH activity, increased PDK1 expression, and PDK inhibition partially rescues GLN starvation-induced ROS and cell death. Finally, we show that combining GLN starvation with pro-oxidants selectively kills GLN addicted cells. These data highlight a major role for GLN in maintaining redox balance in cancer cells that lack glucose-dependent anaplerosis.

Cancer cells exhibit abnormal glucose and GLN metabolism to confer growth and survival advantages under restrictive conditions of the tumor environment^1^,^2^,^3^,^4^. GLN is the most abundant amino acid in plasma and is consumed by tumor cells at much greater rates than any other amino acid^5^,^6^,^7^,^8^. Akin to glucose, GLN is a major precursor for ATP synthesis and anaplerosis (replenishment of TCA cycle intermediates). In addition to satisfying bioenergetic needs, GLN has many other important functions in proliferating cells, including synthesis of proteins, nucleic acids, lipids, hexosamines, glutathione (GSH), and NADPH^6^. Not surprisingly, many cells rapidly consume GLN during growth, and some oncogenes stimulate GLN metabolism in the context of cell proliferation^9^,^10^. Moreover, many cancer cell lines depend on access to exogenous GLN for survival, a phenomenon known as GLN addiction. However, the basis for this selective dependency on GLN for survival is poorly understood.

ROS are produced in all cells as byproducts of normal cellular processes or are generated as signaling messengers^11^. Oncogene activation or inactivation of tumor suppressors is associated with increased ROS production, which in some cases is critical for cellular transformation^10^,^12^,^13^. However, depending on the overall concentrations, ROS can have diverse downstream effects. Low or moderate levels of ROS promote and sustain oncogenic properties of cancer cells^14^,^15^,^16^, whereas excessive ROS can cause oxidative damage to lipids, proteins, and DNA, eventually resulting in cell death^17^. Therefore, efficient management of ROS levels is crucial for cancer cell survival. Several studies have shown that cancer cells can be selectively killed by small molecule inducers of oxidative stress^18^,^19^,^20^, underscoring the importance of fine-tuning of ROS levels for cancer cell survival. Cancer cells compensate for increased ROS production by engaging ROS-scavenging systems involving antioxidants such as GSH and Thioredoxin (Trx)^21^. Scavenging of ROS results in oxidation of GSH and Trx, which are recycled back to their reduced forms by GSH reductase and Trx reductase, respectively, in an NADPH dependent manner^11^. Therefore NADPH is indispensible for cellular antioxidant activity. Both GSH and NADPH can be synthesized from GLN^22^, and recent studies have implicated GLN as a key factor in ROS homeostasis in cancer cells^9^,^23^.

Here, using diverse human cancer cell lines, we show that cells that do not rely on GLN for survival are also independent of GLN for ROS homeostasis. Dependency on GLN for survival correlates with a preference for GLN as an essential component of antioxidant capacity. We found that GLN starvation-induced ROS elevation causes increased glucose uptake in GLN addicted cells. However, tracing glucose carbons by metabolic labeling indicated that GLN addicted cells do not utilize glucose for anaplerosis, and indeed GLN starvation almost completely abolishes glucose metabolism via the TCA cycle in these cells, despite the increased glucose uptake. In contrast, GLN independent cells are able to use both glucose and GLN in anaplerosis, and in the absence of GLN can compensate by utilizing glucose-dependent anaplerosis. However, inhibition of the mitochondrial pyruvate transporter in GLN independent cells leads to GSH depletion and oxidative stress, rendering them GLN addicted. Moreover, GLN starvation increases PDK1 expression and activity, as observed by increased levels of phosphorylated (inactivated) PDH, only in GLN addicted cells, accounting for reduced glucose oxidation via the TCA cycle. In line with this, inhibition of PDK activity rescues GLN starvation-induced oxidative stress and cell death. Finally, we show that GLN starvation synergizes with the pro-oxidant small molecule piperlongumine in effective killing of GLN addicted tumor cells.

## Results

### GLN starvation-induced cell death correlates with increased ROS levels

**Figure 1:**
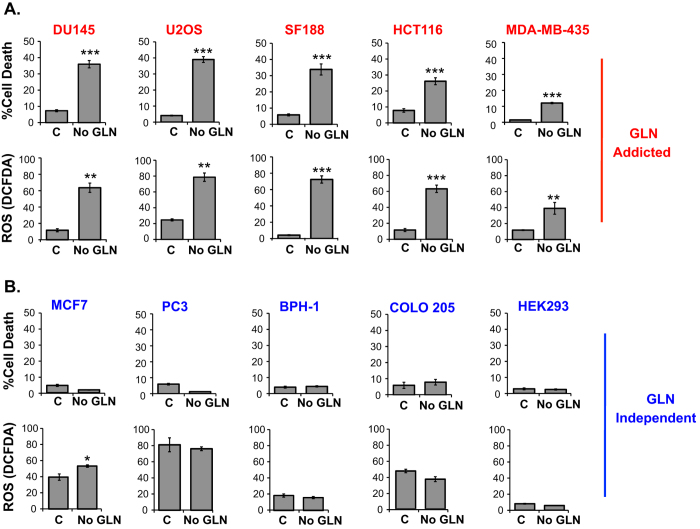
GLN starvation-induced cell death correlates with increased ROS levels. Cells were categorized as (**A**) GLN addicted (red font) and (**B**) GLN independent (blue font) based on their sensitivity to GLN starvation. Cell death was measured after 72 hours of GLN starvation (except for DU145 and SF188 cells, in which cell death was measured after 48 hours of GLN starvation due to their extreme sensitivity to GLN depletion) by PI staining followed by flow cytometry. ROS levels were measured after 48 hours GLN starvation (24 hours for DU145 and SF188 cells due to their extreme sensitivity to GLN depletion) by DCFDA staining followed by flow cytometry. Data are the average ± SD of three independent cultures. *P < 0.05, **P < 0.005, ***P < 0.001.

Because GLN is a precursor for GSH and NADPH antioxidant synthesis and effective ROS management is vital for cancer cell survival, we hypothesized that GLN starvation-induced cell death correlates with increased ROS. To test this we measured ROS and cell death after GLN starvation using the general ROS indicator DCFDA and propidium iodide (PI) staining, respectively, in different human cancer cell lines. GLN starvation induced significant cell death only in cell lines that exhibited increased ROS levels after GLN withdrawal (Fig. 1A). On the other hand, ROS levels were not affected in cell lines that did not require GLN for survival (Fig. 1B). We also measured ROS and cell death at an earlier time point and found that ROS is induced after 20 hours of GLN starvation, whereas no significant cell death was observed at this time point (Fig. S1), suggesting that GLN starvation induces ROS prior to cell death. We designated cells sensitive to GLN withdrawal as GLN addicted (shown in red fonts in Fig. 1A), and insensitive cells as GLN independent (shown in blue font in Fig. 1B). Thus, we define GLN addiction as dependency of cells on GLN for survival. These results demonstrate a strong correlation between GLN starvation-induced cell death and ROS elevation.

To determine whether genomic differences could explain GLN dependence versus independence in the cell line panel, we evaluated mutational and copy number alterations in publically available genomic datasets from the Cancer Cell Line Encyclopedia (CCLE)^24^, and from published literature. We failed to identify consistent differences between 4 GLN dependent and 3 GLN independent cell lines, although 3 of 4 GLN dependent but none of the independent cell lines showed CTNNB1 alterations (Fig. S2). While CTNNB1 alterations have previously been associated with sensitivity to GLN^25^,^26^, the significance of this observation remains to be determined.

### GLN starvation leads to enhanced depletion of antioxidant pools in GLN addicted cells

**Figure 2:**
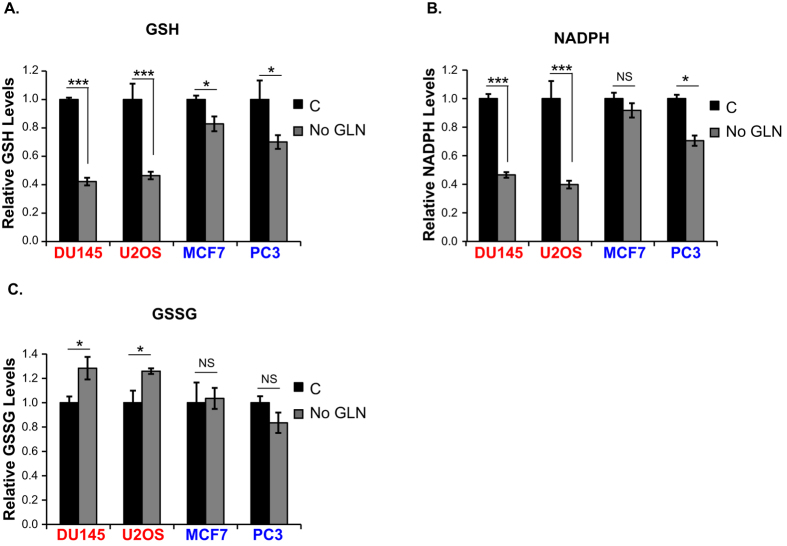
GLN starvation severely reduces antioxidant pools in GLN addicted cells. GSH **(A)**, NADPH (**B**), and GSSG (**C**) levels were measured in GLN addicted (DU145, U2OS) and GLN independent (MCF7, PC3) cells after 20 hours of GLN starvation. Data are the average ± SD of three independent cultures. *P < 0.05, ***P < 0.001.

GLN is a precursor for production of GSH and NADPH, which are critical for managing ROS levels. Because GLN withdrawal increased ROS only in the GLN addicted cells, we predicted that depletion of GSH and NADPH pools should be more prominent in GLN addicted cells after GLN withdrawal. Indeed, levels of both GSH and NADPH were reduced by more than 50% in GLN addicted DU145 and U2OS cell lines after 24 hours of GLN starvation (Figs 2A and S3A), whereas only modest or non-significant changes were observed in the GLN independent cell lines, MCF7 and PC3 (Fig. 2B). Notably, addition of glutamate (GLU) significantly increased both GSH and NADPH levels in DU145 and U2OS cells (Fig. S3B,C). We also measured oxidized glutathione (GSSG) levels in the same cell lysates that were used in the GSH measurements, and found that GLN starvation significantly increases GSSG levels only in GLN addicted cells (Fig. 2C), indicating that GLN independent cells maintain sufficient levels of GSH and NADPH even after GLN starvation. Collectively, these findings suggest that GLN addicted cells rely predominantly on GLN for GSH and NADPH generation, whereas GLN independent cells likely utilize other precursors in addition to GLN.

### GSH or GLU inhibit GLN starvation-induced cell death and ROS elevation

**Figure 3:**
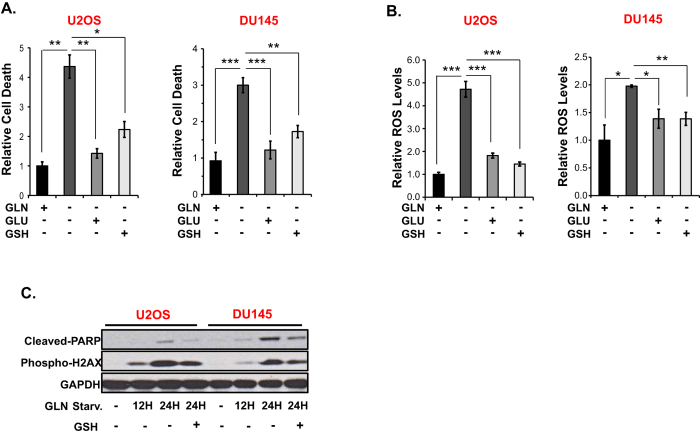
GLU and GSH inhibit GLN starvation-induced cell death and ROS increase. Effect of GLU (5 mM) and GSH (3 mM) on GLN starvation-induced (**A**) cell death and (**B**) ROS in GLN addicted DU145 and U2OS cells. Cell death and ROS were measured after 48 hours and 30 hours of GLN starvation respectively by PI or DCFDA staining. Data are the average ± SD of three independent cultures. *P < 0.05, **P < 0.005, ***P < 0.001. (**C**) Western blot analysis of cleaved PARP and phospho-H2AX levels in GLN addicted DU145 and U2OS cells after 12–24 hours GLN starvation. 3 mM GSH was used to assess its effect on GLN-starvation-induced induction of cleaved PARP and phospho-H2AX. Blots were cropped for clarity.

To determine the contribution of the observed GLN starvation-induced ROS increase to cell death, we tested the ability of GLU or the antioxidant GSH to rescue GLN starvation-induced cell death in two GLN addicted cell lines, U2OS and DU145. GLU is the downstream product of GLN and can be directly used in GSH synthesis; alternatively, after conversion to α-KG, it can enter the TCA cycle to supply NADPH-producing pathways. Both GLU and GSH addition inhibited GLN starvation-induced cell death and ROS increases (Fig. 3A,B) similar to previously reported findings^27^,^28^. The mechanism by which addition of extracellular GSH to the medium reduces ROS remains unknown, but likely represents breakdown of GSH to glutamate in the medium, which could then be taken up by SLC1A3 as reported^29^,^30^. However, we cannot rule out direct uptake of intact GSH into cells, as has also been described^31^,^32^. Addition of the antioxidant NAC also inhibited GLN starvation-induced cell death and ROS in DU145 cells (Fig. S4A). To complement these findings, we assessed GLN starvation-induced cell death and oxidative stress by Western blot analysis of the apoptotic cell death marker, cleaved-PARP^33^, and, since oxidative stress causes DNA damage that is associated with increased H2AX phosphorylation^34^, using phospho-H2AX levels. Consistent with the above results, GLN starvation markedly increased cleaved-PARP and phospho-H2AX in the GLN addicted cell lines U2OS and DU145, which were suppressed by addition of GSH (Fig. 3C). In marked contrast, GLN starvation failed to significantly increase either cleaved-PARP or phospho-H2AX levels in the two GLN independent cell lines, PC3 and BPH-1 (Fig. S4B), indicating that GLN starvation causes only minimal oxidative damage in these cells. We used etoposide as a positive control for a functional DNA damage response, as it causes DNA strand breaks and leads to increased H2AX phosphorylation^35^,^36^. Etoposide treatment increased phospho-H2AX levels in both PC3 and BPH-1 cells, ensuring that there are no defects in H2AX phosphorylation in these cells. Note that in addition to GSH synthesis, GLN is also required for nucleotide synthesis^37^. However, GLN starvation-induced phospho-H2AX upregulation can be inhibited by addition of both GLU and GSH, which are not known to be precursors for nucleotide synthesis. Together, these results indicate that GLN starvation induces cell death predominantly by increasing cellular ROS and oxidative stress.

### GLN starvation increases glucose uptake in GLN addicted cells in a ROS-dependent manner

**Figure 4:**
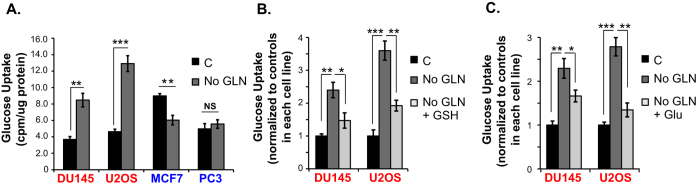
GLN starvation increases glucose uptake in GLN addicted cells in a ROS dependent manner. (**A**) ^14^C-glucose uptake by GLN addicted (DU145, U2OS) and GLN independent (MCF7, PC3) cells was measured after 18–20 hours of GLN starvation. ^14^C-glucose uptake by GLN addicted DU145 and U2OS cells was measured after 20 hours of GLN starvation with/without 3 mM GSH (**B**) or 5 mM GLU. (**C**) Data are average ± SD of 3 independent cultures. *P < 0.05, **P < 0.005, ***P < 0.001.

Because antioxidant levels were less prominently reduced after GLN withdrawal in GLN independent versus dependent cells, we wondered whether in the absence of GLN, the former might differentially increase glucose uptake to generate more NADPH, as glucose is a major source of NADPH production via the pentose phosphate pathway^22^. This would be predicted to increase recycling of GSSG back to GSH to counterbalance oxidative stress. To test this we measured glucose uptake in cells under GLN withdrawal. Unexpectedly, while glucose uptake was reduced or unchanged in GLN independent MCF7 and PC3 cells, respectively, it was dramatically elevated in GLN addicted DU145 and U2OS cells 24 hours after GLN withdrawal (Fig. 4A). We then tested if increased glucose uptake in the GLN addicted cells is due to increased ROS after GLN withdrawal. Addition of GSH (Fig. 4B) or GLU (Fig. 4C) into the culture medium almost completely blocked GLN starvation-induced glucose uptake in both DU145 and U2OS cells. Therefore rather than mitigating ROS generation, these findings suggest that elevation of glucose uptake is downstream of GLN starvation-induced ROS, and that GLN dependent cells are characterized by increased ROS-induced glucose uptake under GLN withdrawal.

### GLN addicted cells are defective in utilizing glucose for the TCA cycle under GLN starvation despite increased glucose uptake

**Figure 5:**
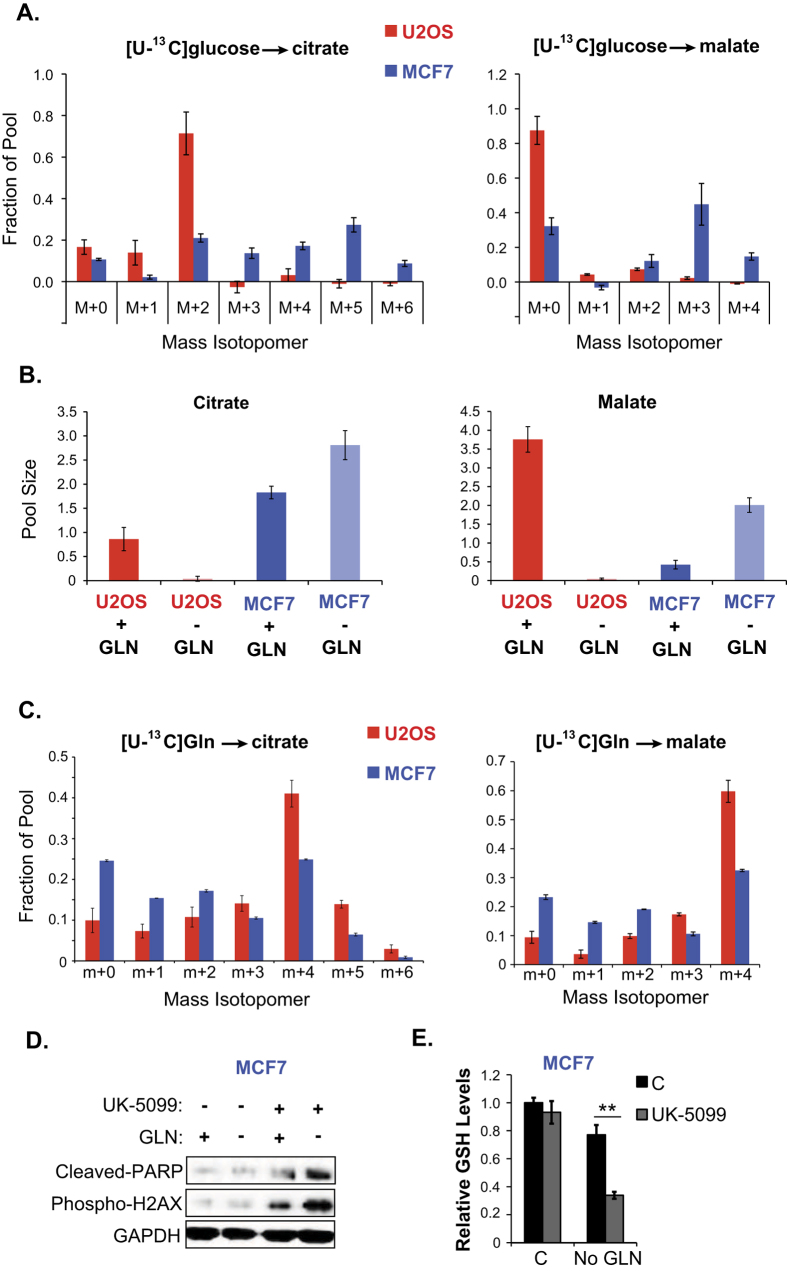
GLN addicted cells lack glucose-dependent anaplerosis in the absence of GLN. (**A**) Comparison of mass isotopomers of citrate and malate in U2OS and MCF7 cells cultured with [U-^13^C]glucose and unlabeled GLN for 8 hours. (**B**) Analysis of citrate and malate pools in U2OS and MCF7 cells GLN starved for 24 hours and labeled with [U-^13^C_6_]glucose for 8 hours. (**C**) Comparison of mass isotopomers of citrate and malate in U2OS and MCF7 cells cultured with [U-^13^C5]GLN and unlabeled glucose for 16 hours. (**D**) Analysis of glucose, lactate, GLN, and GLU levels in culture media of U2OS and MCF7 cells. (**E**) Western blot analysis of cleaved PARP and phospho-H2AX levels in MCF7 cells +/− GLN starved for 24 hours and treated with 100 μM of mitochondrial pyruvate transporter inhibitor UK-5099. (**F**) Effect of 100 μM UK-5099 on GSH levels in MCF7 cells +/− GLN starved for 24 hours. Blots were cropped for clarity. High contrast was used for clarity and original blots are given in Fig. S7. Data are average ± SD of 3 independent cultures. ^**^P < 0.005.

Increased versus unchanged glucose uptake by GLN addicted cells and GLN independent cells, respectively, under GLN starvation prompted us to investigate how these cells utilize glucose in the TCA cycle. We therefore compared glucose metabolism in GLN addicted U2OS versus GLN independent MCF7 cells in the presence or absence of GLN. To do this we performed a metabolic labeling study using uniformly labeled ^13^C-glucose, and found marked differences in how these cells utilize glucose in the TCA cycle. When GLN was present, GLN addicted cells displayed prominent m + 2 labeling in citrate (~70% of the total pool), indicating that the bulk of anaplerotic flux to oxaloacetic acid (OAA) came from an unlabeled substrate (i.e. GLN) (Fig. 5A). In contrast, GLN-independent cells exhibited a broader distribution of ^13^C labeling, indicating enrichment of the OAA pool from glucose. This labeling pattern is consistent with pyruvate carboxylation as a source of anaplerosis^38^. Malate labeling revealed a similar pattern as observed with citrate (Figs 5A and S5A). When GLN was deprived from GLN addicted cells, pools of the TCA cycle intermediates citrate and malate were almost completely depleted (Fig. 5B). By contrast, GLN independent cells were able to maintain both citrate and malate pools in the absence of GLN (Fig. 5B). Metabolic labeling of U2OS and MCF7 cells using uniformly labeled ^13^C-GLN revealed that the GLN addicted U2OS cells predominantly display evidence of the first turn of the TCA cycle, resulting in a large m + 4 fraction in malate and citrate and indicating a large anaplerotic flux relative to total TCA cycle activity (Fig. 5C). In contrast, the MCF7 cells show a broader distribution of labeling in malate and citrate (Fig. 5C), similar to results of the ^13^C-glucose labeling studies. We did not see a difference in intracellular lactate labeling from ^13^C-GLN in either cell line (Fig. S5B). Both cell lines consumed glucose and glutamine, and secreted lactate (Fig. 5D). These data suggest that GLN independent cells are somehow able to compensate for GLN deprivation to maintain pools of TCA cycle intermediates, whereas GLN addicted cells cannot.

A previous study reported that GLN independent cells overcome the limitations of interrupted GLN metabolism by employing pyruvate carboxylase-mediated pyruvate entry into TCA cycle^38^. Results of the above metabolic labeling studies with GLN independent MCF7 cells also imply that the ability to use glucose as an anaplerotic precursor to support mitochondrial metabolism in the absence of GLN may be advantageous to cells in preventing GLN starvation-induced oxidative stress and cell death. To test this idea, we inhibited mitochondrial pyruvate transport in MCF7 cells using the specific inhibitor UK-5099 and evaluated the contribution of defects in pyruvate channeling into the TCA cycle to GLN starvation-induced oxidative stress and cell death. Treatment with UK-5099 markedly increased oxidative stress and cell death in the absence of GLN, as apparent from markedly increased phospho-H2AX and cleaved PARP levels (Fig. 5E). In parallel, UK-5099 treatment caused a significant reduction in GSH production in the absence of GLN (Fig. 5F). Together, these results support a model whereby GLN independent cells have adopted a mechanism that can alternate between utilization of different nutrients depending on their availability, which allows them to control their redox state and thus survive. In stark contrast, GLN addicted cells cannot utilize glucose in the TCA cycle when GLN is absent, leading to a block in mitochondrial metabolism and increased cellular ROS.

### GLN starvation increases PDK1 expression and reduces PDH activity in GLN addicted cells

**Figure 6:**
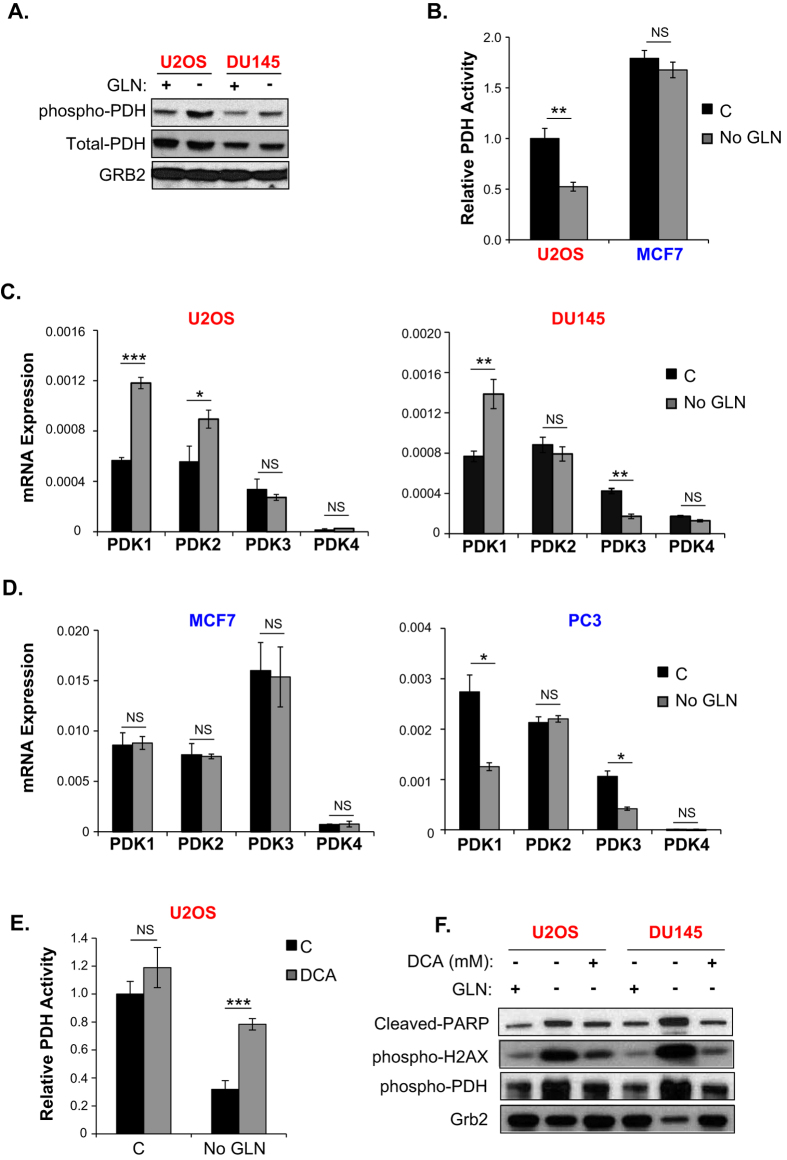
GLN starvation reduces PDH activity and increases PDK1 in the GLN addicted cells. (**A**) Western blot analysis of phospho-PDH levels in GLN addicted (U2OS, DU145) cells +/− GLN starved for 20 hours. Total PDH and Grb2 were used as loading controls. Blots were cropped for clarity. (**B**) PDH activity assay in U2OS and MCF7 cells +/− 20H GLN starvation. (**C**) Relative mRNA levels of four PDK isoforms in GLN addicted U2OS and DU145 cells and (**D**) in GLN independent MCF7 and PC3 cells +/− 20 H GLN starvation. (**E**) Effect of 2 mM dichloroacetate (DCA) on PDH activity in U2OS cells +/− 20 H GLN starvation. (**F**) Western blot analysis of cleaved-PARP, phospho-H2AX as cell death and oxidative stress markers, and phopsho-PDH in U2OS cells treated with indicated concentrations of DCA +/− 20 hour GLN starvation. Grb2 was used as loading control. Blots were cropped for clarity. Full length blots are given in Fig. S7. Data are average ± SD of 3 independent cultures. *P < 0.05, **P < 0.005, ***P < 0.001.

Entry of glucose-derived carbons into the TCA cycle is regulated by the mitochondrial pyruvate dehydrogenase (PDH) complex, which catalyzes conversion of pyruvate to acetyl coenzyme A. Phosphorylation of PDH by pyruvate dehydrogenase kinases at the E1 subunit renders the PDH complex inactive^39^,^40^. Since GLN addicted cells are defective in utilizing glucose in the TCA cycle in the absence of GLN, we predicted that PDH activity may be reduced by GLN starvation in GLN addicted cells. Using a phospho-specific PDH E1 subunit antibody, we analyzed the phospho-PDH levels in GLN addicted and GLN independent cells in the presence and absence of GLN by Western Blots. As shown in Fig. 6A, phospho-PDH levels increase only in the GLN addicted U2OS and DU145 cells upon GLN starvation, but not in the GLN independent MCF7 and PC3 cells (Fig. S6). We also compared the PDH enzyme activity in U2OS and MCF7 cells with or without GLN starvation. Consistent with increased phospho-PDH levels, PDH enzyme activity was significantly reduced (~50%) in GLN addicted U2OS cells compared to GLN independent MCF7 cells upon GLN starvation (Fig. 6B). Of note, U2OS cells exhibited ~2-fold lower PDH activity than MCF7 cells, which may explain why incorporation of glucose carbons into TCA cycle intermediates is lower in the U2OS cells as compared to MCF7 cells under GLN replete conditions. Since phosphorylation of PDH is regulated by PDK, we compared the expression levels of PDK isoforms in GLN addicted cells and GLN independent cells. PDK has four known isoforms with varying expression levels depending on the tissue and context^41^. Of the four isoforms, PDK1 expression was consistently increased in GLN addicted U2OS and DU145 cells upon GLN starvation, whereas in GLN independent MCF7 and PC3 cells it was unchanged or actually reduced (Fig. 6C,D). We then used dicholoroacetate (DCA), a well characterized PDK inhibitor^42^, in GLN addicted U2OS cells to determine whether inhibition of PDK increases PDH activity and rescues GLN starvation-induced oxidative stress and cell death. DCA treatment increased PDH activity (Fig. 6E) and partially reduced ROS levels and cell death based on phospho-H2AX and cleaved-PARP levels (Fig. 6F). Together these results suggest that inhibition of PDH activity by GLN starvation further reduces the entry of glucose-derived pyruvate carbons into the TCA cycle.

### Combining GLN starvation with oxidative stress inducing agents effectively kills GLN addicted cells

**Figure 7:**
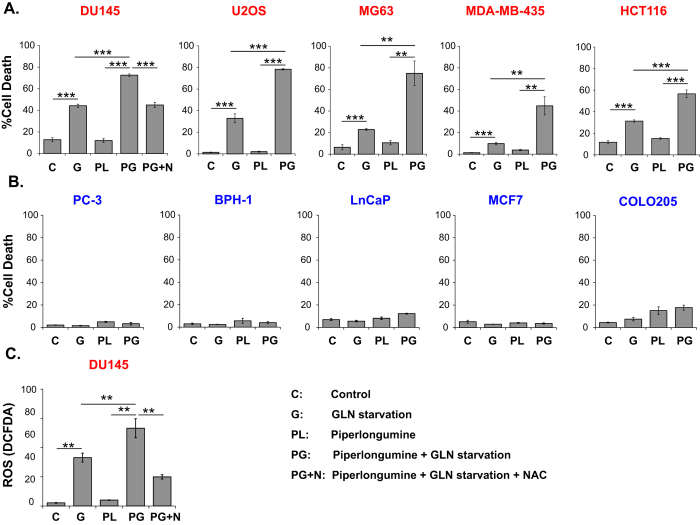
GLN starvation synergizes with oxidative stress-inducing agents. Effect of 5 μM PL on GLN starvation-induced cell death in (**A**) GLN addicted and (**B**) GLN independent cells. (**C**) Effect of PL on GLN starvation-induced ROS levels in DU145 cells. Cell death was measured after 48 hours of GLN starvation by PI staining. ROS were measured after 30 hours GLN starvation by DCFDA staining. 5 mM NAC was added where indicated. Data are average ± SD of 3 independent cultures. **P < 0.005, ***P < 0.001.

The above studies clearly demonstrate that GLN addicted cells depend on GLN to maintain sufficient pools of antioxidants and to manage ROS levels. Accordingly, we predicted that treating GLN addicted cells with pro-oxidant reagents while depriving them from a critical antioxidant source, GLN, would efficiently kill them by exacerbating oxidative stress. One such pro-oxidant reagent is piperlongumine (PL), which was previously shown to increase oxidative stress^19^,^43^. We therefore tested the efficiency of PL treatment alone or in combination with GLN starvation in inducing cell death in GLN addicted versus GLN independent human cancer cell lines. PL treatment alone at a low (sublethal) concentration did not cause significant cell death in any of the lines when the cells were supplemented with GLN; however, the same concentration induced marked cell death in GLN addicted cells in the absence of GLN (Fig. 7A). In contrast, and consistent with the results shown above, PL treatment did not synergize with GLN starvation in killing GLN independent cells (Fig. 7B). ROS levels also correlated with cell death as shown in GLN addicted DU145 cells, in which PL treatment amplified GLN starvation-induced ROS levels (Fig. 7C). These findings indicate that GLN independent cells can use other means to reduce ROS in the absence of GLN, whereas GLN addicted cells predominantly depend on GLN to cope with elevated ROS.

## Discussion

Many cancer cells depend on GLN for survival (so-called GLN addiction), but why dependence on GLN for survival varies so much among different tumor cell types remains elusive. Here we show that GLN addiction in human cancer cell lines is related to mechanisms of antioxidant defense. Our findings uncover a mechanism of GLN starvation-induced cell death whereby GLN starvation first leads to increased ROS levels in GLN addicted cells which in turn leads to increased glucose uptake. GLN independent cells appear to escape this oxidative stress crisis and ensuing cell death by evading the initial ROS increase when GLN is absent. Metabolic labeling studies using ^13^C-glucose indicate a dichotomy between GLN addicted and independent cells in how these cells metabolize glucose in the TCA cycle. GLN addicted cells exhibit glucose independent anaplerosis when supplied with GLN, whereas GLN starvation almost completely inhibits glucose metabolism via the TCA cycle. In contrast, GLN independent cells are able to utilize glucose for anaplerosis, and can compensate for GLN loss by replenishing TCA cycle pools with glucose-derived pyruvate. This in turn enables them to maintain critical antioxidant levels even in the absence of GLN.

GLN was initially viewed as a nitrogen and carbon source in cancer cells for the synthesis of macromolecules and for energy production, and therefore GLN starvation-induced cell death was associated with energetic crisis. Yuneva *et al.* demonstrated that c-Myc transformation renders cells GLN addicted, but GLN starvation-induced cell death was shown to be independent of GSH depletion and ROS increases in their studies^44^. However, a later study reported GSH depletion and ROS elevation upon GLN starvation in Myc dependent human cancer cell lines^9^. Therefore recent reviews of cancer cell metabolism have begun to implicate GLN as an essential source of GSH synthesis, and GLN withdrawal-induced cell death has been linked to depletion of GSH pools and increased ROS in human cancer cell lines^22^,^45^. Here we show that GLN starvation-induced GSH depletion and ROS elevation are not universal features of human cancer cells, and establish a correlation between GLN-starvation-induced cell death and ROS elevation. GLN starvation depletes antioxidant pools (GSH and NADPH) only in GLN addicted cells, suggesting that some cells do not rely solely on GLN and are able compensate by perhaps using other sources such as glucose to maintain their antioxidant pools. Accordingly, we expected GLN independent cells to take up more glucose under GLN starvation as compared to the GLN addicted cells. However, we found instead that GLN addicted cells markedly increase glucose uptake, whereas GLN independent cells maintain or even decrease glucose uptake when GLN is withdrawn. Both GSH and GLU block the increase in glucose uptake in the absence of GLN, indicating that GLN addicted cells increase glucose uptake as an attempt to compensate for GLN loss. Exactly how GLN addicted cells increase their glucose uptake in the absence of GLN remains unknown. Glucose can be metabolized via the pentose phosphate pathway and/or the TCA cycle to produce NADPH and GLU (the precursor of GSH), respectively. Hence, it is paradoxical that GLN addicted cells cannot maintain their antioxidant pools and suppress ROS despite the surge of glucose in the absence of GLN. One possibility is the inability of the GLN addicted cells to utilize glucose efficiently. Although GLN independent cells do not increase glucose uptake with GLN starvation, they may be able to shunt glucose to the appropriate pathways to compensate for GLN depletion. For instance, pyruvate carboxylase has been shown to confer GLN independence by facilitating OAA formation from glucose-derived pyruvate^38^. OAA then condenses with acetyl-CoA to produce citrate, which can then be used in GSH synthesis after conversion to α-KG, and eventually to GLU. Our findings support this premise, as GLN independent cells appear to use a combination of glucose-derived pyruvate and other sources of carbon (e.g. GLN) for anaplerosis. When GLN is absent, GLN addicted cells cannot metabolize glucose via the TCA cycle, presumably because their inability to produce abundant quantities of OAA in the absence of GLN prevents them from oxidizing glucose-derived acetyl-CoA. In sharp contrast, GLN independent cells compensate by increasing pyruvate carboxylation, and inhibition of mitochondrial pyruvate transporters by the specific inhibitor UK-5099 leads to increased oxidative stress, cell death, and depletion of GSH, rendering GLN independent cells GLN addicted. Note that NADPH generation from GLN involves the malic enzyme pathway. Conversion of malate to pyruvate by malic enzyme is accompanied by reduction of NADP+ to NADPH, which can be inferred by assessing incorporation of GLN carbons into lactate. In our GLN labeling experiment we did not see a difference in lactate labeling by GLN in either GLN addicted U2OS cells or GLN independent MCF7 cells. It is likely that when GLN is present, both GLN addicted and independent cells generate NADPH from GLN via the malic enzyme pathway. However, when GLN is absent, GLN independent cells continue to generate NADPH via this pathway since they are able to generate malate from glucose.

Another explanation for why GLN addicted cells cannot utilize glucose in the TCA cycle is the deficient PDH activity. Phosphorylation of PDK inactivates PDH and inhibits the entry of glucose-derived carbons into the TCA cycle^39^,^40^. Indeed, GLN starvation increases phosho-PDH levels and decreases PDH activity only in the GLN addicted cells. In line with these findings, GLN starvation increases specifically and consistently the PDK1 isoform in the GLN addicted cells and pharmacological inhibition of PDK activity rescues GLN starvation-induced oxidative stress and cell death. These data corroborate that GLN addicted cells evolved to adopt mechanisms that do not favor glucose oxidation via the TCA cycle at the expense of dependency on a single precursor to maintain antioxidant pools.

Several studies have demonstrated the vulnerability of cancer cells to ROS-inducing agents^18^,^19^,^20^,^46^. Our results show that GLN starvation, if combined with the ROS-inducing compound PL at a concentration that is sub-toxic when GLN is present, triggers massive cell death in GLN addicted cells in the absence of GLN. Interestingly, when GLN is present PL does not cause ROS increases at the concentrations used; however, it enhances ROS levels in the absence of GLN. Both cell death and ROS induction is inhibited by NAC, indicating that the observed effects are ROS-dependent.

It is important to note that avidity of GLN consumption *in vivo* appears to vary widely among different types of tumors. For instance, Marin-Valencia *et al.* showed that GLN is dispensable for glioblastoma tumor growth *in vivo*^47^. In contrast, other studies demonstrated GLN consumption in tumors as a fuel source *in vivo*^7^,^8^. Therefore our studies potentially provide insights into cell-autonomous effects that may dictate glutamine addiction or independence in different tumor types. In conclusion, our findings suggest that GLN addicted cells depend on GLN to cope with both endogenous and exogenous oxidative stress, highlighting the potential for combined treatment using GLN depletion and oxidants as an efficient strategy for the treatment of GLN dependent tumors. However, since GLN independent cells appear to be resistant to this treatment, the successful execution of such an approach will likely depend on identification of biomarkers for GLN addiction Although our findings are based on *in vitro* culture models, we believe that they provide important insights into tumor cell metabolism in the context of GLN dependence versus independence.

## Materials and Methods

### Cell Culture and Inhibitors

All cell lines, except HCT116, were cultured in high glucose (25 mM) DMEM (Sigma) containing 2 mM glutamine and supplemented with 10% FBS (Sigma) and 1% penicillin/streptomycin (Invitrogen). HCT116 cells were maintained in McCoy’s 5 A medium (Sigma). 1 μg/mL Insuling (Sigma) was added into culture medium for MCF7 cells. For glutamine starvation experiments, cells were seeded in regular media and the next day it was replaced by high glucose DMEM without glutamine and pyruvate (Gibco) supplemented with 10% dialysed FBS (Invitrogen) and 1% penicillin/streptomycin. 2–4 mM glutamine was added where stated. UK-5099 and DCA were purchased from Sigma.

### Cell Death and ROS Measurements

Cells were seeded in 6-well plates in triplicates. For cell death measurements, after indicated treatments both detached and attached cells were pooled, centrifuged, resuspended in cold PBS containing 1 μg/mL propidium iodide (PI) (Molecular Probes), and analyzed immediately using FACSCalibur flow cytometer (BD Biosciences) in FL-3 channel. ROS levels were measured using the general ROS indicator CM-H2DCFDA (5-(and-6)-chloromethyl-2′,7′-dichlorodihydrofluorescein diacetate, acetyl ester) (Molecular Probes). After indicated treatments, cells were incubated with 5 μM DCFDA for 40 minutes. Medium was removed and the cells were washed with PBS. After trypsinyzing, cells were centrifuged and resuspended in PBS containing 1 μg/mL PI, and analyzed immediately by flow cytometry using FL-1 channel for DCFDA and FL-3 channel for PI fluorescence.

### GSH and NADPH Assays

Intracellular reduced or oxidized glutathione levels were measured using a commercial kit (BioVision). ~1.0 × 10^6^ cells were seeded in 6-cm dishes, and after indicated treatments cells were washed twice with ice-cold PBS, and lysed with 100 μL of GSH assay buffer and GSH was measured according to the manufacturer’s protocol. Intracellular NADPH levels were measured using previously described enzymatic cycling methods^48^. 1.0 × 10^6^ cells were plated in 6-cm dishes in triplicates, after treatments the cells were lysed in 150 μL of extraction buffer (20 mM nicotinamide, 20 mM NaHCO_3_, 100 mM Na_2_CO_3_), passed several times through a 25-gauge needle and centrifuged. To destroy NADP+, 100 μL of the supernatant was incubated at 60 °C for 30 min. 160 μL of NADP-cycling buffer (100 mM Tris-HCl pH 8.0, 0.5 mM thiazolyl blue, 2 mM phenazine ethosulfate, 5 mM EDTA) containing 1.3 U of G6PD was added to a 96-well plate containing 20 μL of the cell extract, and the plate was incubated for 1 minute in the dark at 30 °C. Next, 20 μL of 10 mM glucose 6-phosphate was added into each well, and the change in absorbance at 570 nm was measured every 30 s for 4 min at 30 °C with a Tecan microplate reader.

### ^14^C-Glucose Uptake

^14^C-Glucose Uptake was measured using a method similar to ^14^C-Glutamine Uptake assays as described before^49^,^50^ with modifications. 2–3 × 10^5^ cells were seeded on 6-well plates in triplicates and treated as described in the main text. The media was removed and the cells were overlaid with 800 μL of glucose-free DMEM, incubated at 37 ^ο^C for 10–15 minutes. 2 μL (0.2 μCi/μL) of D-[U-^14^C_6_]glucose (Perkin Elmer) was added into each well and incubated at 37 ^ο^C for 2 minutes. After washing 3 times with 1 mL PBS, 250 μL of lysis buffer (0.2% SDS, 0.2 N NaOH) was added on the cells and incubated for 30 minutes at room temperature, transferred into eppendorf tubes and incubated in a heating block at 60 ^ο^C for another 20 minutes. 25 μL of 1 N HCl was added into each tube to neutralize NaOH. 200 μL of the lysate was transferred into scintillation vials containing 6 mL scintillation liquid (Scintisafe Econo, Fisher Scientific), and the total radioactivity was determined using a β-scintillation counter (Perkin Elmer).

### PDH Activity Assay

PDH activity was measured using a commercial kit (BioVision) according to kit instructions. Results were normalized to protein concentrations measured in the lysates.

### Antibodies

Sources of antibodies included, cleaved Parp, Grb2 (BD Biosciences), phospho-H2AX, GAPDH, Akt, phospho-PDH, total PDH (Cell Signaling).

### Metabolic Labeling With Stable Isotopes and Analysis of Metabolites by GC/MS

For metabolic labeling, DMEM powder lacking glucose, glutamine and pyruvate supplemented with 10% dialyzed FBS and 1% penicillin/streptomycin was used. Cells were first grown in regular DMEM on 10 cm dishes in triplicates to ~80% confluence and after rinsing 2 times with PBS, cells were overlaid with media containing 10 mM D-[U-^13^C_6_]glucose (Sigma) with/without 2 mM GLN and incubated for 8 hours. For ^13^C-GLN labeling cells were seeded and treated as described for glucose labeling except that labeling media contained 10 mM unlabeled glucose and 4 mM [U-^13^C_5_]-GLN and cells were labeled for 16 hours. nCells were then rinsed twice with ice-cold PBS and lysed with three freeze/thaw cycles in 50% ice-cold methanol. The lysates were centrifuged to remove precipitated protein, then evaporated and derivatized by trimethylsilylation (Tri-Sil HTP reagent, Thermo). Three μL of the derivatized material were injected onto an Agilent 6970 gas chromatograph equipped with a fused silica capillary GC column (30 m length, 0.25 mm diameter) and networked to an Agilent 5973 Mass Selective Detector. The abundance of the following ions was monitored: m/z 245–249 for fumarate; m/z 335–339 for malate; and m/z 465–471 for citrate. The measured distribution of mass isotopomers was corrected for natural abundance of ^13^C 51.

### Statistical analysis

Statistical significance was determined using a two-tailed Student’s t-test.

## Additional Information

**How to cite this article**: Cetinbas, N. M. *et al.* Glucose-dependent anaplerosis in cancer cells is required for cellular redox balance in the absence of glutamine. *Sci. Rep.* **6**, 32606; doi: 10.1038/srep32606 (2016).

## Supplementary Material

Supplementary Information
