# Supplementary material for: Glucose-dependent anaplerosis in cancer cells is required for cellular redox balance in the absence of glutamine
Source: Sci Rep. 2016 Sep 8;6:32606. doi: 10.1038/srep32606 (PMC5015067; doi:10.1038/srep32606)

Glucose-dependent anaplerosis in cancer cells is required for cellular redox balance in the absence of glutamine

Naniye Mallı Cetinbas, Jessica Sudderth, Robert C. Harris, Aysun Cebeci, Gian L. Negri, Ömer H. Yılmaz, Ralph J. DeBerardinis, and Poul H. Sorensen

Suppl. Figure 1. GLN starvation induces ROS before cell death. ROS and cell death in U2OS and DU145 cells were measured after 20 hours of GLN starvation by DCFDA and PI staining respectively. Data are the average ± SD of three independent cultures. $^{**}P < 0.005$, $^{***}P < 0.001$.

Suppl. Figure 2. Genomic analysis of GLN dependent vs independent cell lines. Mutational analysis was based on manual inspection of publically available data from the Cancer Cell Line Encyclopedia (CCLE) and the literature1, 2.Amplifications and deep deletions were defined based on GISTIC scores, >1 and <-1, respectively. Cell lines are shown across the top of the image, while gene names are listed along the right border.

Suppl. Figure 3. GLN starvation depletes antioxidant pools in GLN addicted cells. A. NADPH/NADP ratio was measured in GLN addicted U2Os cells after 8, 16, and 24 hours of GLN starvation. Effect of GLU (5 mM) on (B) GSH levels (C) NADPH levels in GLN addicted DU145 and U2OS cells after 20 hours of GLN starvation. Data are the average ± SD of three independent cultures. $^{*}P < 0.05$, $^{**}P < 0.005$, $^{***}P < 0.001$.

Suppl. Figure 4. GLN starvation induces cell death by increasing cellular ROS levels. A. Effect of NAC (3 mM) on GLN starvation-induced cell death and ROS in DU145 cells. Cell death and ROS were measured after 48 hours and 30 hours of GLN starvation respectively by PI or DCFDA staining. 5 μM NAC was used where indicated. Data are the average ± SD of three independent cultures. B. Western blot analysis of cleaved PARP and phospho-H2AX levels in GLN independent PC3 and BPH-1 cells after 24 hours GLN starvation. 10 μM etoposide was used as positive control for phospho-H2AX induction. $^{**}P < 0.005$.

Suppl. Figure 5. Metabolite tracing in 13C-labeled cells. A. Metabolic flux charts showing fate of 13C-glucose and 13C-GLN in glucose-dependent anaplerosis or GLN-dependent anaplerosis. B. Comparison of mass isotopomers of intracellular lactate in U2OS and MCF7 cells cultured with [U-13C]GLN and unlabeled glucose for 16 hours. Data average ± SD of three independent cultures.

Suppl. Figure 6. GLN starvation does not increase phosphor-PDH levels in GLN independent cells. Western blot analysis of phospho-PDH levels in GLN independent (MCF7, PC3) cells +/- GLN starved for 20 hours. Total PDH and Grb2 were used as loading controls.

Suppl. Figure 7. Full length blots of the main figures 5E and 6F. In figure 6E phospho-PDH blot was ran in a separate gel using the same amount of lysates as used for the rest of the blots shown in this figure.

Supplemental Information References:

1. Dan S, Okamura M, Seki M, et al. Correlating phosphatidylinositol 3-kinase inhibitor efficacy with signaling pathway status: in silico and biological evaluations. Cancer research 2010; 70(12): 4982-94.

2. Vlietstra RJ, van Alewijk DC, Hermans KG, van Steenbrugge GJ, Trapman J. Frequent inactivation of PTEN in prostate cancer cell lines and xenografts. Cancer research 1998; 58(13): 2720-3.

Suppl. Figure-1 (Cetinbas)

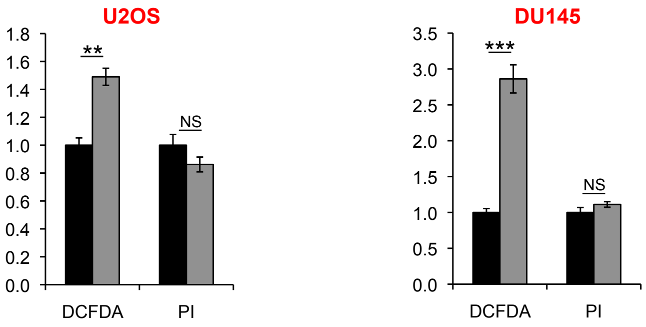

Suppl. Figure-2 (Cetinbas)

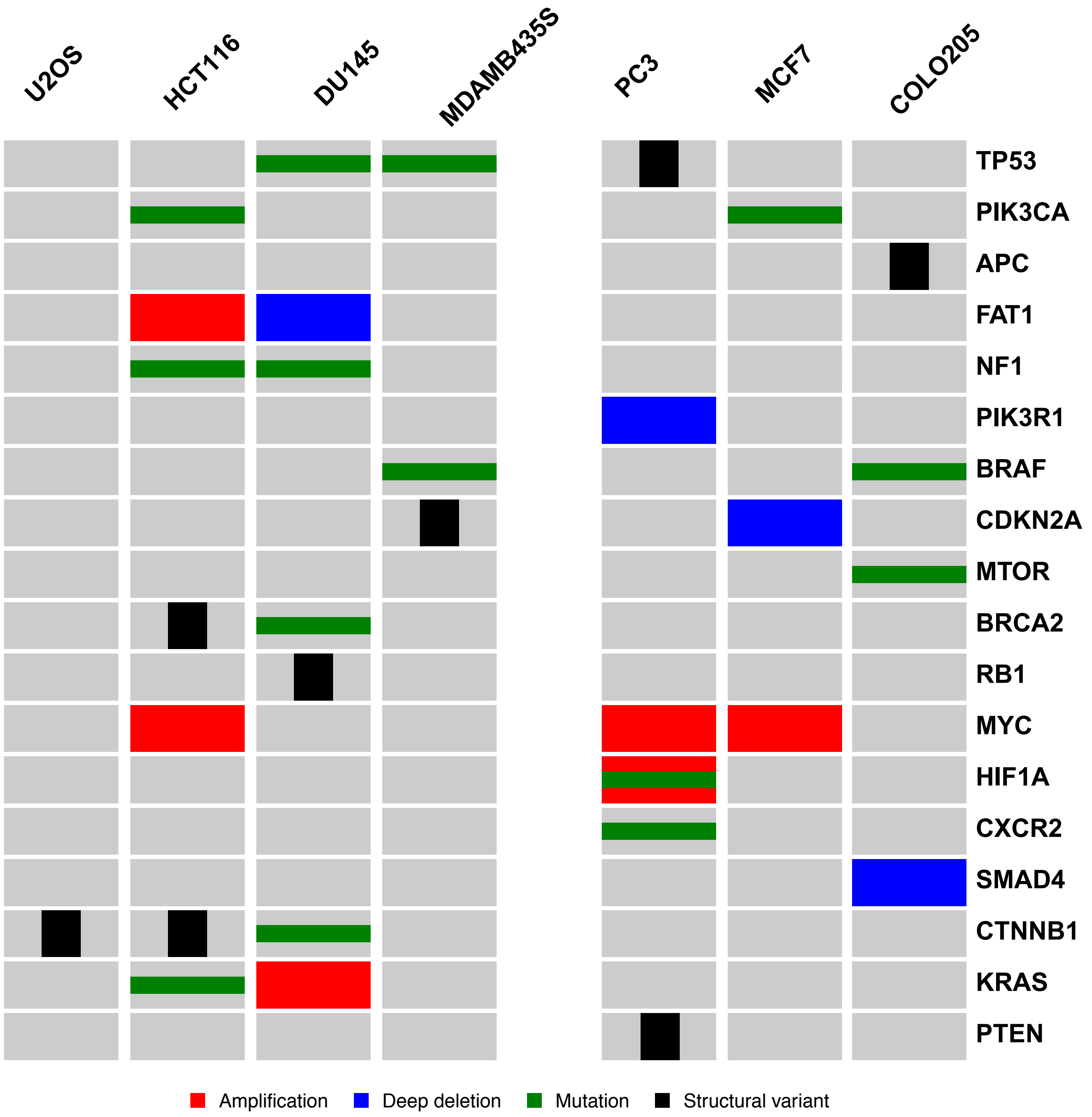

Suppl. Figure-3 (Cetinbas)

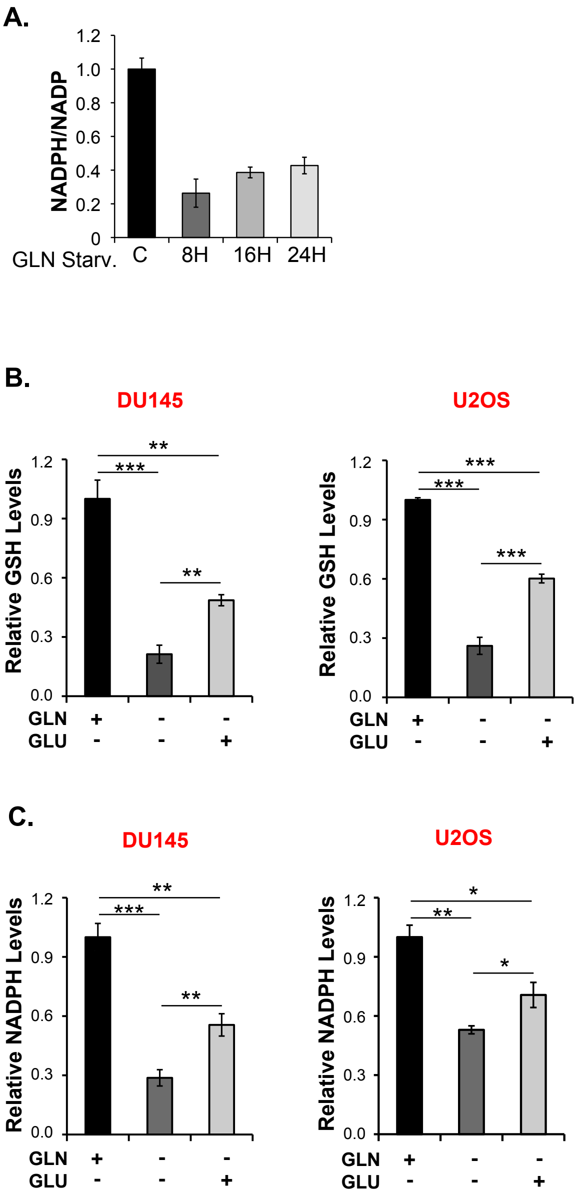

Suppl. Figure-4 (Cetinbas)

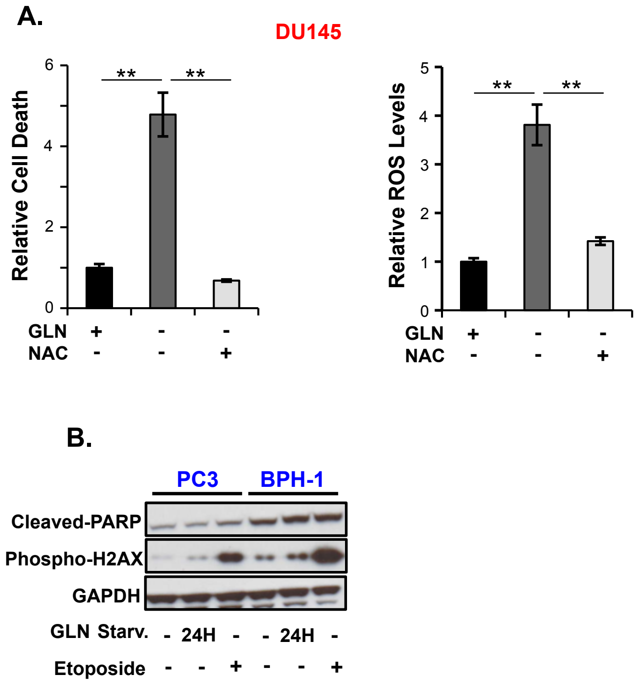

Suppl. Figure-5 (Cetinbas)

A.

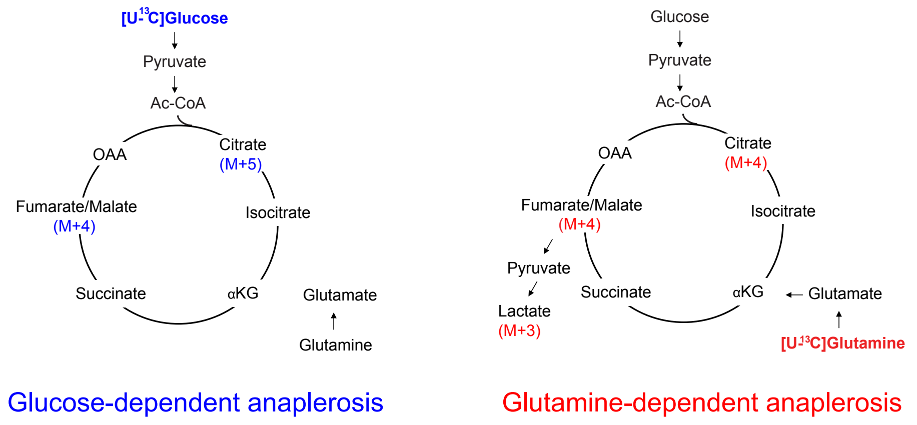

B.

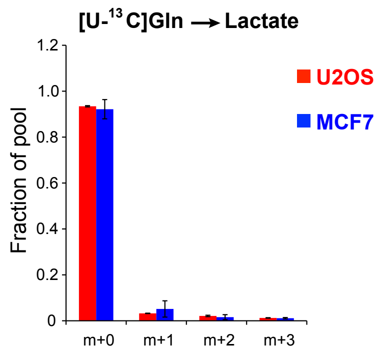

Suppl. Figure-6 (Cetinbas)

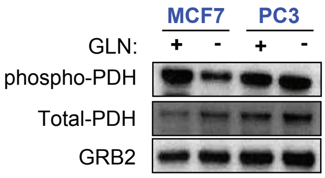

Figure 6F.

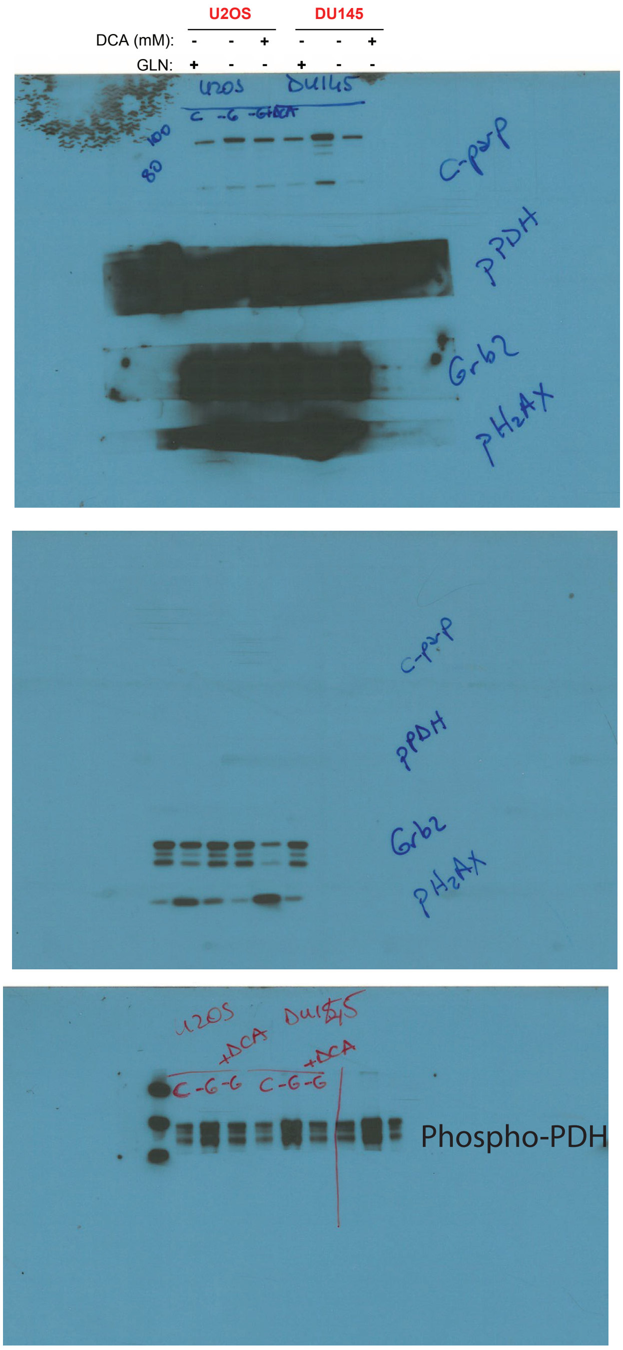

Suppl. Figure 7.

Figure 5E.

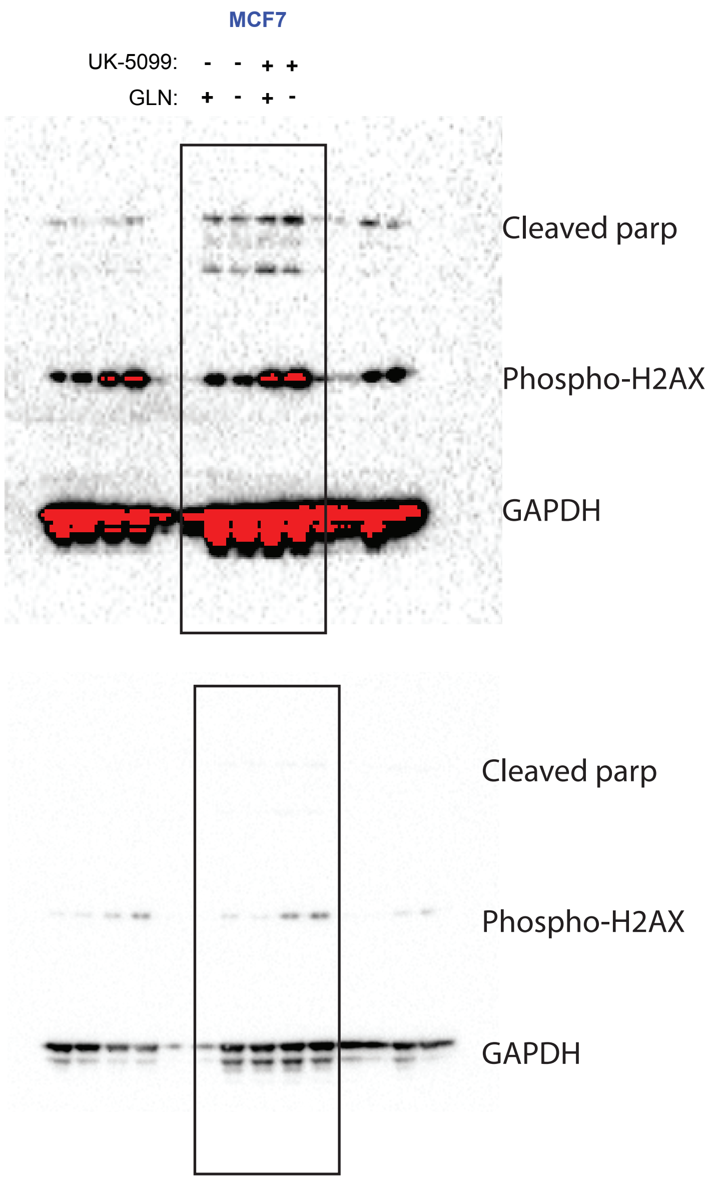

Supplement: Supplementary Information [file srep32606-s1.pdf]
